# Removal of phytotoxins in filter sand used for drinking water treatment

**DOI:** 10.1016/j.watres.2021.117610

**Published:** 2021-10-15

**Authors:** Natasa Skrbic Mrkajic, Jawameer R. Hama, Bjarne W. Strobel, Hans Chr.B. Hansen, Lars Holm Rasmussen, Ann-Katrin Pedersen, Sarah C.B. Christensen, Mathilde J. Hedegaard

**Affiliations:** a Greater Copenhagen Utility HOFOR, Parkstien 10, 2450, Copenhagen, Denmark; b Department of Plant and Environmental Sciences, University of Copenhagen, Thorvaldsensvej 40, 1871 Frederiksberg, Denmark; c Department of Technology, University College Copenhagen, Sigurdsgade 26, 2200 Copenhagen, Denmark

**Keywords:** Biological rapid sand filters, Carcinogens, Emerging contaminantes, Groundwater, Natural toxins, Water quality

## Abstract

Phytotoxins - toxins produced by plants – are contaminants with the potential to impair drinking water quality. They encompass a large group of toxic, partially persistent compounds that have been detected in seepage waters and in shallow wells used for drinking water production. If phytotoxins enter wells used for drinking water production, it is essential to know if the drinking water treatment processes will remove them from the water phase. However, it is currently unknown whether phytotoxins remain stable during traditional groundwater treatment using sand filters as the main treatment process. The objective of this study is to investigate removal potential of phytotoxins in biological sand filters and to asses if the removal potential is similar at different waterworks.

Microcosms were set up with filter sand and drinking water collected at different groundwater-based waterworks. To be able to monitor phytotoxin removal ptaquiloside, caudatoside, gramine, sparteine, jacobine N-oxide, senecionine N-oxide and caffeine were applied at initial concentrations of 300 µg L^-1^, which is approx. two orders of magnitude higher than currently detected in environment, but expected to cover extreme environmental conditions. Removal was monitored over a period of 14 days. Despite the high initial concentration, all filter sands removed ptaquiloside and caudatoside completely from the water phase and at waterworks where pellet softening was implemented (pH 8.4) prior to rapid sand filtration, complete removal occurred within the first 30 min. All filter sands removed gramine and sparteine, primarily by a biological process, while jacobine N-oxide, senecionine N-oxide and caffeine were recalcitrant in the filter sands. During degradation of ptaquiloside and caudatoside we observed formation and subsequent removal of degradation products pterosin B and A. Filter sands with the highest removal potential were characterised by high contents of deposited iron and manganese oxides and hence large specific surface areas. Difference between bacterial communities investigated by 16S rRNA gene analyses did not explain different removal in the filter sands.

All five investigated filter sands showed similar degradation patterns regardless of water chemistry and waterworks of origin. In drinking water treatment systems biological sand filters might therefore remove phytotoxin contaminants such as ptaquiloside, caudatoside, gramine, sparteine, while for other compounds e.g. jacobine N-oxide, senecionine N-oxide further investigations involving more advanced treatment options are needed.

## Highlights

• First study to investigate fate of phytotoxins in drinking water treatment.
• Biological sand filters removed bracken toxins and certain alkaloids.
• Pyrrolizidine alkaloids showed recalcitrant character in sand filters.
• Further investigation involving more advanced treatment are needed.

## Graphical abstract

**Figure:**
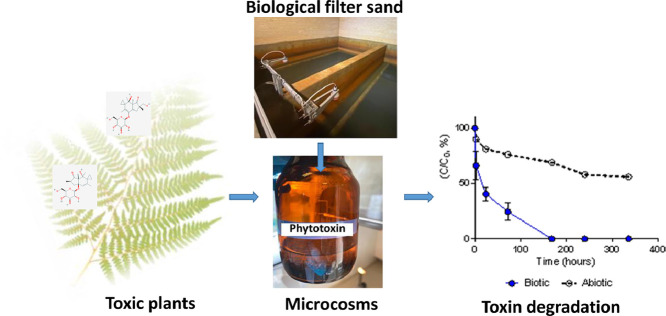

**List of abbreviations**
**BD**: Below limit of detection
**BET**: Brunauer–Emmett–Teller
**BR**: Biological removal
**CAU**: Caudatoside
**Cond**: Conductivity
**DNA**: Deoxyribonucleic acid
**FA**: Formic acid
**FNU**: Formazin Nephelometric Unit
**GRAM**: Gramine
**HPLC-MS**: High Performance Liquid chromatography–mass spectrometry
**Kow**: Octanol-water partition coefficient
**LOD**: Limit of detection
**LOQ**: Limit of quantification
**MRM**: Multiple reaction monitoring
**n.a**: Not available
**NVOC**: Non-volatile organic carbon
**pKa**: Acid dissociation constant
**PMOC**: Persistent and mobile organic compounds
**PTA**: Ptaquiloside
**PtA**: Pterosin A
**PtB**: Pterosin B
**PTFE**: Polytetrafluoroethylene
**SM**: Supplementary material
**SPAR**: Sparteine
**TOC**: Total organic carbon
**TQD**: Triple Quadrupole Mass Spectrometer
**TR**: Total removal
**WHO**: World Health Organization
**WWTPs**: Wastewater treatment plants

## 1 Introduction

Groundwater is used as a drinking water source worldwide (IWA, 2014). In Denmark, groundwater is the primary source of drinking water with < 1% coming from other sources, while in Europe, on average, 70% of the drinking water supply is based on groundwater (IWA, 2014; Navarrete et al., 2008). Groundwater pollutants encompass a wide range of anthropogenic chemicals such as pesticides, pharmaceuticals and household care products (Thomaidis et al., 2012), which have already attracted much attention due to their wide occurrence. However, hazardous compounds can also have a natural origin. Phytotoxins are unexplored contaminants originating from plants and are polar mobile organic compounds (PMOCs), which are neither monitored, treated nor regulated.

*In silico* analyses suggest that a large number of phytotoxins are mobile and persistent in the environment, and should be included in environmental monitoring and risk assessment (Günthardt et al., 2018). Recently, the presence of a broader variety of phytotoxins has been confirmed in surface and seepage water (Clauson-Kaas et al., 2016; Günthardt et al., 2020; Hama, 2020; Nanusha et al., 2020; Kisielius et al., 2020a; Skrbic et al., 2021). In many cases, concentrations of these compounds exceeded the threshold of toxicological concern for drinking water (Nanusha et al., 2020). Moreover, presence of phytotoxins in water bodies might contribute to complex mixture toxicities (Bucheli, 2014) that could jeopardize water quality. While natural toxins such as cyanotoxins are well known drinking water contaminants (guideline maximum value of 1 µg L^-1^ in drinking water for Microcystin-LR) (WHO, 2003), knowledge on phytotoxins occurrence in drinking water is very limited.

A group of well-studied phytotoxins are norsesquiterpene glycosides produced by bracken ferns, which are known for their carcinogenic properties and toxin production in large quantities (IARC, 2018; Kisielius, 2020). The bracken toxins ptaquiloside and caudatoside are highly water soluble and mobile in the environment (Table 1). Ptaquiloside can undergo acid as well as alkaline hydrolysis and the rate of hydrolysis is strongly pH dependant (Ayala-Luis et. al., 2006; Wu et al., 2021). Ptaquiloside, as well as the main product of hydrolysis (pterosin B), can be microbially degraded under environmental conditions in soils (Skourti-Stathaki et al., 2016). Ptaquiloside is found in surface water with reported concentrations up to 2.2 µg L^-1^ (Clauson-Kaas et al., 2016) and in seepage water up to 0.35 µg L^-1^ (Skrbic et al., 2020). Caudatoside is more polar than ptaquiloside and has recently been detected in surface waters in Denmark (Kisielius, 2020) and shallow water wells (0.75 µg L^-1^) (Skrbic et al., 2021). The detected concentrations exceed the maximum estimated tolerable concentration of ptaquiloside (estimated to 0.002 µg L^-1^ (Rasmussen, 2003)) in drinking water.

**Table 1:** Physicochemical properties of norsesquiterpene glycosides and alkaloids investigated in the study.

	Ptaquiloside	Caudatoside	Jacobine N-oxide	Senecionine N-oxide	Gramine	Sparteine	Caffeine
Molecular structure							
CAS number^a^	**87625-62-5**	**-**	**38710-25**	**13268-67-2**	**87-52-5**	**90-39-1**	**58-08-2**
Molecular formula	**C_20_H_30_O_8_**	**C_21_H_32_O_9_**	**C_18_H_25_NO_7_**	**C_18_H_25_NO_6_**	**C_11_H_14_N_2_**	**C_15_H_26_N_2_**	**C_8_H_10_N_4_O_2_**
Water solubility [g L^-1^]^b^	**2.0**	**8.0**	**10.2**	**3.8**	**32.2**	**3.0**	**2.6**
log Kow^b^	**-0.95**	**-1.87**	**-0.5**	**0.3**	**1.5**	**2.7**	**0.16**
pKa^c^	**-**	**-**	**2.8**^c^**, 12.4**^d^	**2.8**^c^**, 12.4**^d^	**7.9**	**12**	**10.4**

EC_50_[mg L^−1^] e	**-**	**-**	**-**	**-**	**6.03**^e^	**28.6**^e^	**551**^f^
LC_50_ [mg L^−1^] g	**881**	**3576**	**-**	**-**	**0.2**	**1.9**	**119**

a Structures made in ChemDraw V.16.0

b Calculation with EPISuite v4.0

c ACD/Percepta 2016.2

d Chemicalize

e Concentration causing 50% immobility towards *Daphnia magna* 48 h (Griffiths et al., 2021)

f (Brackett et al., 1990)

g ECOSAR_data of Lethal concentration 50 (LC_50_) towards *Daphnia magna* 48 h (Günthardt et al., 2018). LC_50_ values for jacobine N-oxide and senecionine N-oxide are not available, however LC_50_ for jacobine and senecionine are 67 and 50 mg/L respectively, which are corresponding free base PAs.

Alkaloids is the most abundant class of phytotoxins, which encompass more than 12,000 compounds (Bucheli, 2014; Crozier et al., 2008). Pyrrolizidine alkaloids are considered defence chemicals used by plants against insects and herbivores (Van Egmond, 2004). The pyrrolizidine alkaloids jacobine N-oxide and senecionine N-oxide (Table 1) have been measured in a surface water ponds at concentrations up to 47 and 17 µg L^-1^, respectively, while about 10 times lower concentrations have been detected in stream water (Kisielius et al., 2020a). The N-oxide forms of the pyrrolizidine alkaloids have been more frequently detected than pyrrolizidine alkaloids free base forms in the environment (Günthardt et al., 2020; Hama and Strobel, 2020a).

Sparteine is a tetracyclic quinolizidine alkaloid, while gramine is a bicyclic alkaloid including an indole moiety (Table 1). Both are abundant in *Fabaceae* (*Leguminosae*) plant family (Boschin and Resta 2013). Sparteine and gramine were detected in soil pore water at concentrations exceeding 0.1 µg L^-1^ in areas cropped with lupins (*Lupinus* spp.) (Hama and Strobel, 2020b). Caffeine has been detected in groundwater e.g. up to a concentration of 0.68 µg L^-1^ in Italy (Castiglioni et al., 2018). Even though the origin of caffeine in groundwater is probably of anthropogenic origin, it indicates that structurally similar natural alkaloids such as pyrrolizidine and quinolizidine alkaloids may be sufficiently persistent to reach groundwater reservoirs.

There is a paucity of ecotoxicity data on selected phytotoxins (Bucheli, 2014). However, the measured concentrations of selected phytotoxins in water resources may exceed the threshold of toxicological concern level for drinking water (Mons et al., 2013). The World Health Organisation (WHO) and the International Agency for Research on Cancer classifies bracken fern as possible carcinogenic to humans (Group 2B) while ptaquiloside is considered not classifiable as to its carcinogenicity to humans (Group 3) due to lack of experimental data. There are no environmental guidelines for pyrrolizidine alkaloids in water resources, but e.g. WHO recommends minimum exposure and EFSA recommends a maximum daily exposure of 23.7 ng kg^−1^ body weight (Kisielius et al., 2020a).

Most phytotoxins are polar, with low Kow and high water solubility, and some exist as cations (Günthardt et al., 2018). Hence, they are mobile in the environment, and may contaminate groundwater (Table 1). Groundwater-based drinking water treatment is usually a simple process, and in Denmark it consists of aeration of anaerobic groundwater followed by rapid biological sand filtration. During aeration, the water reaches an oxygen concentration of 8-10 mg L^-1^, while volatile compounds such as methane and hydrogen sulphide are stripped off. Oxygen is needed for oxidation of ammonium, Fe(II) and Mn(II) – taking place in rapid sand filters (Mouchet, 1992). Rapid sand filters typically have a retention time between 7 and 10 min (Winter et al., 2003), but can be up to 1 hour. During filtration, Mn(IV) and Fe(III) form metal hydroxides and oxides (from now on called metal oxides) (Teunissen et al., 2008), which deposit in the filter material, yielding metal oxide coatings on filter sand (quartz). Mature sand filter material is therefore rich in Fe and Mn oxides and has higher specific surface areas than uncoated sand (Sharma et al., 2002). The biological rapid sand filters also harbour microbiological processes, such as ammonium oxidation. The microbiological community in the filter sand is affected by the inlet water chemistry and varies between different waterworks (Albers et al., 2015; Palomo et al., 2016).

Micropollutants can be removed from drinking water by processes such as ozonation followed by biological activated carbon filtration (Van der Hoek et al., 1999) or granular activated carbon (Heijman et al., 2002). However, if micropollutants can be handled in simple biological rapid sand filters, it would be of large practical and commercial interest, since biological rapid sand filters are inexpensive and sustainable (Godskesen et al., 2011). Thus, Benner et al. (2013) suggested to utilize sand filters as treatment technology for removal of micropollutants.

Even though rapid sand filters are not designed to eliminate organic micropollutants, their potential to remove pesticides has been demonstrated (Hedegaard and Albrechtsen, 2014), and full scale rapid sand filters at a groundwater-based waterworks removed 46% of the herbicide mecoprop (initial concentration 0.046 µg L^-1^) within a contact time of 8 min (Hedegaard et al., 2014). Lab-scale investigations with filter sand have shown that microbiological degradation of pesticides such as bentazone led to complete mineralization (Hedegaard et al., 2019). However, pesticide degradation can depend on certain groups of bacteria, such as methanotrophs (Hedegaard et al., 2020). Since the microbial communities of rapid sand filters depends on inlet water chemistry and age (Albers et al., 2015; Palomo et al., 2016), the removal potential of micropollutants might differ between waterworks.

It is unknown whether phytotoxins would remain stable during traditional groundwater treatment using sand filters as the main treatment process, and thus potentially impair drinking water quality. To our knowledge, phytotoxin removal potential in the existing drinking water treatment has still not been investigated. The objective of this study is to investigate the removal potential and mechanism of terpenoid and alkaloid phytotoxins in filter sand from biological rapid sand filters and to assess if the removal rates are similar at different waterworks. Here we focus on three different phytotoxin classes, which are toxic, mobile and recently detected in water resources: norsesquiterpene glycosides (ptaquiloside and caudatoside), quinolizidine alkaloids (sparteine and gramine) and pyrrolizidine alkaloids (jacobine N-oxide and senecionine N-oxide). In addition, we include the methylxanthine alkaloid caffeine, a persistent compound commonly found in groundwater, that also serves as a tracer compound in this study.

To be able to screen several different sand filters and compounds we performed these investigations as batch experiments. In order to ensure reliable monitoring of the removal and to test a worst-case scenario, phytotoxins were applied at initial concentrations of 300 µg L^-1^. To better understand the mechanisms behind phytotoxin removal processes we performed physicochemical and DNA analyses of the filter sand.

## 2 Materials and methods

### 2.1 Chemicals and analytical standards

Methanol (MeOH) (MS grade), formic acid (FA) (MS grade) and PTFE membrane filter (0.2 μm) were purchased from Sigma-Aldrich (Darmstadt, Germany). Cellulose acetate filters (0.22 µm) were purchased from Frisenette (Knebel, Denmark). Analytical standards of ptaquiloside and caudatoside were prepared from bracken plant material by the method described by Kisielius et al. (2020b). Analytical standards of gramine, sparteine and caffeine were purchased from Sigma-Aldrich (Darmstadt, Germany), and jacobine, jacobine N-oxide, senecionine, and senecionine N-oxide were purchased from Phytolab (Vestenbergsgreuth, Germany). MilliQ water (resistivity 18.2 Mohm × cm, TOC less than 1 µg L^-1^) was produced in-house with a type I ultrapure water purification system from ELGA-Veolia LabWater (High Wycombe, UK).

### 2.2 Analytical procedures

Two different analytical methods were used for the sample analyses. Quantification of ptaquiloside and caudatoside were performed by Agilent 1260 Infinity HPLC System equipped with Agilent 6130 Single Quadrupole mass spectrometer by the method described by Kisielius et al. (2020b). For quantification of alkaloids, samples were analysed on a Waters Acquity UPLC I-Class module, equipped with Acquity UPLC HSS C18 column by the method described by Hama and Strobel (2019, 2020b). More details about the analytical procedures and MS settings is provided in supplementary material (SM), Table S1 and Table S2.

### 2.3 Collection of filter sand and water

The investigation included filter sand collected at four waterworks in Denmark, including five biological rapid sand filters. At these waterworks the treatment processes consist of aeration of anaerobic groundwater followed by filtration in primary and in two cases, secondary rapid sand filters (Table 2). After filtration, the treated water is led to clean water tanks and distributed to consumers.

**Table 2:** Investigated waterworks and water treatment steps.

Waterworks	Production* (m^3^ year^−1^)	Softening	Aeration	EBCT of primary sand filters# (min)	EBCT of secondary sand filters# (min)	UV	Age of sand filters (years)
Slangerup	**7,300,000**	**-**	**Coplator**	**22**	**+**	**-**	**> 15**
Hvidovre	**202,000**	**-**	**Stairs**	**72**	**-**	**-**	**> 20**
Brøndby new	**587,000**⁎⁎	**Pellet reactors**	**Coplator**	**13**	**-**	**+**	**3**
Brøndby old	**587,000**⁎⁎	**Pellet reactors**	**Coplator**	**13**	**-**	**+**	**> 20**
Regnemark	**11,100,000**	**-**	**Stairs**	+	**23**	**-**	**> 50**

⁎ Estimated production in 2020

⁎⁎ Total production at Brøndby waterworks;

# Empty-bed-contact-time of sampled filters;

+ Treatment step is included at the waterworks; - Treatment step not included at the waterworks

Filter sand was collected from the primary sand filters at the waterworks, except at Regnemark waterworks, where the filter sand originated from a secondary filter, since the primary filters contains gravel and stones (Table 2). Regnemark is the largest of the investigated waterworks (production of 11.1 million m^3^ per year) and the filter sand has been undisturbed for approximately 50 years. Hvidovre waterworks treats approximately 200.000 m^3^ per year and has the longest empty-bed-contact-time (72 min) of the investigated rapid sand filters (Table 2). Slangerup waterworks is the only investigated waterworks that receives water containing methane (Table 3). At Brøndby waterworks, the water is softened in pellet reactors by addition of NaOH and subsequent precipitation of CaCO_3_ prior to aeration and rapid sand filtration. Hence, the inlet water to the filters is characterized by elevated pH (pH 8.4) and less water hardness (Table 3). Two different sand filters were sampled at Brøndby waterworks: an old filter established before the softening process was implemented, and hence the sand had a similar metal oxide coating as traditional sand filters, while the other, a new filter was implemented along with the softening process, thus having less metal oxide coating.

**Table 3:** Water quality data for each of the investigated waterworks.

Waterworks		Slangerup		Hvidovre		Brøndby			Regnemark	
	Units	inlet	outlet	inlet	outlet	inlet	outlet new	outlet old	inlet	outlet
Sampling date	**ddmmyy**	**101219**	**101219**	**180220**	**180220**	**110520**	**110520**	**110520**	**080620**	**080620**
Temperature	**° C**	**-**	**-**	**10.3**	**10.2**	**9.6**	**9.6**	**9.6**	**9.4**	**9.4**
pH	**-**	**7.7**	**7.6**	**7.8**	**7.7**	**8.4**	**8.3**	**8.2**	**7.3**	**7.3**
Cond. at 25°C	**mS m^-1^**	**71**	**81**	**138**	**137**	**97**	**99**	**99**	**100**	**100**
Turbidity	**FNU**	**8.71**	**0.21**	**6.9**	**0.44**	**0.25**	**0.32**	**0.34**	**3.0**	**0.43**
Iron	**mg L^-1^**	**0.88**	**B.D.**	**0.61**	**0.025**	**0.06**	**0.012**	**0.018**	**0.41**	**0.023**
Manganese	**mg L^-1^**	**0.05**	**B.D.**	**0.06**	**B.D.**	**-**	**-**	**-**	**0.03**	**B.D.**
Ammonium	**mg L^-1^**	**0.5**	**B.D.**	**0.12**	**B.D.**	**0.23**	**0.005**	**0.008**	**0.3**	**0.020**
Nitrite	**mg L^-1^**	**B.D.**	**0.002**	**0.01**	**B.D.**	**0.004**	**B.D.**	**B.D.**	**0.02**	**0.002**
Nitrate	**mg L^-1^**	**B.D.**	**1.4**	**3.3**	**3.7**	**1.4**	**2.1**	**2.1**	**1.3**	**2.2**
Oxygen	**mg L^-1^**	**-**	**-**	**10.4**	**9.4**	**12**	**10.5**	**10.3**	**8.3**	**7.6**
Methane	**mg L^-1^**	**0.02**	**B.D.**	**B.D.**	**B.D.**	**B.D.**	**B.D.**	**B.D.**	**B.D.**	**B.D.**
NVOC	**mg L^-1^**	**4.5**	**4.2**	**1.9**	**2.0**	**2.7**	**4.2**	**2.7**	**3.1**	**2.9**

Cond. = conductivity; NVOC = Non-volatile organic carbon; FNU = Formazin Nephelometric Unit; B.D. = below limit of detection

Filter sand was collected from the top 20 cm of the filter bed with a plastic bottle on an extendable shaft, which was previously disinfected with 1% hypochlorite and rinsed with water. The filter sand was transported to the laboratory in a disinfected plastic bucket covered by water from the sand filters on the top. Water was collected before (inlet water) and after (outlet water) biological rapid sand filtration (Table 3) in cleaned bottles rinsed with deionized water and transported to the laboratory while kept at 4 °C.

### 2.4 Experimental set-up (microcosms) and sampling

Within three hours of collecting water and filter sand at the waterworks, 50 g of wet filter material was transferred with a sterilized spoon to 250 mL amber glass bottles, which had previously been acid-washed and heated to 555 °C for 12 h. 50 mL outlet water was added to each bottle, and hence microcosms were set-up. Abiotic controls were prepared with filter sand that was autoclaved three times (20 min, 1 bar and 121 °C) and cooled down before the water was added.

To investigate phytotoxin removal, microcosms were spiked with ptaquiloside, caudatoside, sparteine, gramine, jacobine N-oxide, senecionine N-oxide and caffeine to an initial concentration of 300 µg L^-1^. We investigate pyrrolizidine alkaloids N-oxide forms rather than their free base forms due to the more frequent occurrence of the N-oxide forms in the environment (Günthardt et al., 2020; Hama and Strobel, 2020a). Microcosms were prepared in duplicates for each compound, while abiotic controls were single batches. Microcosms and abiotic controls were kept standing at 8-10 °C in the dark for the study period of 14 days. Microcosms from Slangerup waterworks were only followed for 7 days and only in biotic samples. After spiking microcosms with phytotoxins sampling started, and were collected at time 0 min (C_0_), and after 30 min, 1 h, 3 h, 1 day, 3 days, 7 days, 10 and 14 days. Before sampling the microcosms were gently mixed, the lids were removed and samples were collected with sterile syringes and needles, filtered using 0.2 μm filters and 0.5 mL was transferred to LCMS vials. Final samples were diluted by a factor 2 using 100% MeOH and kept at - 20 °C until analysis, no later than 10 days after the samples were collected. C_0_ samples were used for recovery calculations of biotic and abiotic microcosms in order to evaluate degradation of phytotoxins (concentration in samples divided by the initial concentration (C_0_) in the microcosm). Degradation data were fitted using a first-order model (one phase exponential decay equation) in GraphPad Prism version 7.0 (GraphPad Software, San Diego, California USA). We calculated total removal and biological removal after 7 days. Total Removal was calculated as a residual between the initial concentration (100%) and the measured recovery of phytotoxins. Biological removal was determined as the difference between recovery in biotic and abiotic samples.

### 2.5 Characterisation of filter sand material

Filter sand samples were kept at - 20 °C until physicochemical analyses. We determined particle size distribution, specific surface areas and content of Fe, Mn, C and N in five investigated sand filters (Table 4). More details about the analyses is provided in supplementary material.

**Table 4:** Physicochemical characteristics of five investigated sand filters. “± “ represents standard deviation (n = 2).

Waterworks	Surface area (m² g^-1^)	Fe (mg g^-1^)	Mn (mg g^-1^)	C (%)	N (%)	Particle size distribution (%)				
						> 2 mm	1-2 mm	0.5-1 mm	0.2-0.5 mm	< 0.2 mm
Slangerup	**39.2**	**53.2±0.5**	**10.1±0.0**	**0.70**	**0.08**	**6.3**	**93.0**	**0.6**	**0.2**	**0.1**
Hvidovre	**56.0**	**80.1±0.2**	**20.4±0.2**	**0.67**	**0.09**	**56.5**	**41.0**	**1.3**	**1.0**	**0.2**
Brøndby new	**0.3**	**0.7±0.0**	**0.03±0.0**	**0.21**	**0.02**	**0.5**	**47.4**	**30**	**21**	**1.0**
Brøndby old	**19.7**	**30.4±0.8**	**5.20±0.4**	**0.43**	**0.04**	**1.7**	**73.5**	**23**	**1.6**	**0.5**
Regnemark	**67.9**	**70.7±2.1**	**64.9±0.7**	**1.49**	**0.20**	**5.8**	**82.8**	**9.9**	**1.1**	**0.8**

Filter sand from Hvidovre waterworks contained a high fraction (57%) of large grains (>2 mm) (Table 4). In contrast, the youngest filter sand at Brøndby waterworks (new filter), only contained 0.5% of large particles (>2 mm). Filter sand from Hvidovre and Regnemark waterworks had the largest surface areas and the highest content of Fe and Mn (Table 4). Filter sand from Brøndby waterworks was different between new and old filters, where old filter material had significantly larger surface area and metal oxide content. The old filter at Brøndby waterworks had been in use prior to implementation of pellet softening, and had thus established a metal coating similar to the other waterworks. In contrast, a new filter was implemented along with pellet softening, and since pellet softening is removing Fe(II) and Mn(II) along with other metals, the filter metal oxide coating on the new filter was substantially less developed. The highest C content was measured at Regnemark and Slangerup waterworks.

### 2.6 Filter sand DNA analyses

Filter sand for DNA analyses was immediately stored at -20 °C upon arrival to the laboratory. For two waterworks (Regnemark and Brøndby), DNA analyses of filter sand were performed on sand both before and after the microcosm experiment. DNA extractions, DNA analysis and data analysis were performed at Statens Serum Institut. DNA from filter sand material was extracted by a modified version of the previously described methods (Andersen et al., 2013) using 500 µl wet filter sand material and 1.4 mm ziconium beads. DNA was amplified using four set of primers targeting nuclear ribosomal genes (16S-18S) as described in Krogsgaard et al. (2018). Sequences were mapped using a k-mer-based mapping software (BION) previously described by Ring et al. (2017). The DNA sequencing data generated in this study are publicly available in the European Nucleotide Archive (ENA) and more details about samples and accession numbers are provided in SM (Table S3).

## 3 Results and discussion

### 3.1 Degradation and kinetics of investigated phytotoxins at Regnemark waterworks

**Fig. 1:**
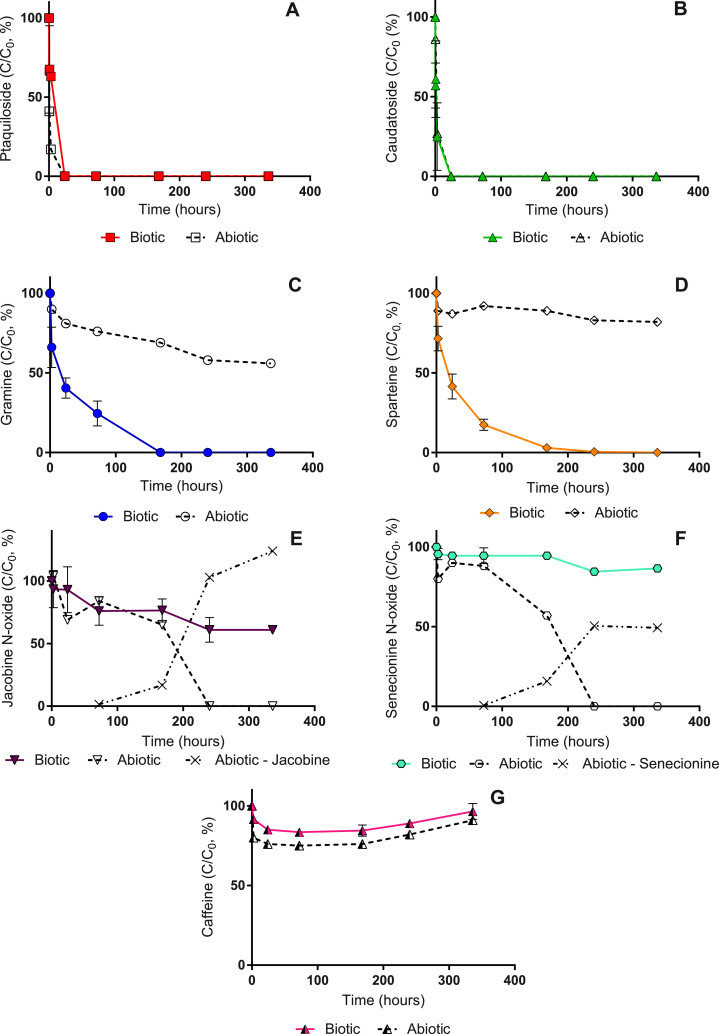
Dissipation of seven phytotoxins in microcosms using sand filter material from Regnemark waterworks over a period of 14 days (at 10 °C). Mean concentration and standard deviation (error bars) are given as percentage of the initial concentration (C_0_, 300 µg L^-1^) in biologically active microcosms (duplicates) and abiotic control (single microcosm). Jacobine and senecionine concentrations are given as percentage of the initial concentration of jacobine N-oxide and senecionine N oxide. ‘Biotic’ refers to microcosms with biologically active filter sand and ‘abiotic’ to microcosms with autoclaved filter sand.

We investigated degradation patterns and kinetics of phytotoxins in filter sand, illustrated by results from Regnemark waterworks (Fig. 1). Similar degradation curves for all other waterworks are available in SM.

The filter sand removed ptaquiloside and caudatoside to below the LOD within 24 h (Fig. 1, A and B). Removal was governed by an abiotic process, since removal was either similar in biologically active microcosms and abiotic controls (caudatoside), or higher in abiotic controls (Fig. 1, A and B). The calculated half-life of ptaquiloside is 291 h (Ayala-Luis et al., 2006), but in contact with filter sand from Regnemark (pH 7.3) we observed the that half-life was 3-4 h, showing that the filter sand accelerated hydrolysis, probably due to the presence of acid metal-oxide coatings on the filter sand.

Biologically active microcosms removed gramine and sparteine substantially faster than abiotic controls (Fig. 1, C and D, Table 5), and removal was thus primarily caused by biological processes. After seven days, we could not detect any gramine and sparteine in biotic samples, while in the abiotic samples they were present even after 14 days in high concentrations (56 and 82% respectively). The pyrrolizidine alkaloids, jacobine N-oxide and senecionine N-oxide were stable in biological microcosms (Fig. 1, E and F), with 60 – 87% recovery after 14 days. Interestingly, filter sand transformed jacobine N-oxide and senecionine N-oxide to jacobine and senecionine (free base form) in abiotic samples after 10 days. Free base form of pyrrolizidine alkaloids are more toxic than the corresponding N-oxide forms (Chou et al., 2003; Wang et al., 2005). We observed a decrease from 60% to 0% for both N-oxide forms while we measured a corresponding increase in free base form. This transformation was also noted in soil degradation experiments and in rat liver and animals gut (Chou et al., 2003). Under oxidative conditions, reduction of the N-oxide to pyrrolizidine alkaloid free base form is inhibited (Wang et al., 2005). The observed transformation in abiotic samples could be due to production of reductive materials during the autoclaving process that reduce N-oxide forms, while the non-autoclaved oxic conditions were maintained in biotic samples and hence N-oxides remained preserved. However, since the biologically active microcosms did not remove jacobine N-oxide and senecionine N-oxide, removal and/or transformation is not relevant for the full-scale system, where rapid sand filters are always biologically active. No significant degradation of caffeine occurred in biotic and abiotic microcosms (Fig. 1, G), and 90% of the initial caffeine was still present after 14 days.

**Table 5:** Total removal (TR) and biological removal (BR) of investigated phytotoxins in five biological sand filters after 7 days (active microcosms in duplicates and abiotic control as single microcosm). Values represent percentages (1-C/C_0_)*100 and “± “ represents standard deviation.

Waterworks	Ptaquiloside		Caudatoside		Jacobine N-oxide		Senecionine N-oxide		Gramine		Sparteine		Caffeine	
	**TR**	**BR**	**TR**	**BR**	**TR**	**BR**	**TR**	**BR**	**TR**	**BR**	**TR**	**BR**	**TR**	**BR**
Slangerup	**84±0**	**n.a.**	**96±0**	**n.a.**	**18±12**	**n.a.**	**7±1**	**n.a.**	**47±0**	**n.a.**	**38±4**	**n.a.**	**5±3**	**n.a.**
Hvidovre	**91±1**	**0**	**100±0**	**-**	**9±11**	**0**	**31±17**	**0**	**40±5**	**1**	**56±3**	**50**	**13±3**	**0**
Brøndby new	**100±0**	**-**	**100±0**	**-**	**17±6**	**0**	**9±1**	**0**	**47±1**	**15**	**52±0**	**27**	**17±0**	**0**
Brøndby old	**100±0**	**-**	**100±0**	**-**	**8±3**	**0**	**9±1**	**0**	**65±0**	**37**	**52±0**	**11**	**15±3**	**5**
Regnemark	**100±0**	**-**	**100±0**	**-**	**24±7**	**0**	**5±1**	**0**	**100±0**	**69**	**97±1**	**86**	**16±2**	**0**

TR = Total removal (%); BR = Biological removal (%); n.a = not available

The filter sand removed a substantial fraction of ptaquiloside, caudatoside, gramine and sparteine from the water phase during the first hour of the experiment (Fig. 1). Therefore, we find the removal relevant compared to the contact time in the filters (13-72 min, Table 2).

### 3.2 Degradation and kinetics for all waterworks

The inlet water chemistry varies between different waterworks (Table 3) and this can influence removal of the investigated compounds, for instance by affecting the composition of the microbiological community in the filter sand (Albers et al., 2015; Palomo et al., 2016). It was therefore investigated how removal of phytotoxins varied between five different sand filters (Table 5; Figure S1-Figure S4), by determining the total removal (TR) and biological removal (BR) in the filter sand.

All sand filters removed between 84 and 100% of the initial ptaquiloside and caudatoside within 7 days. This removal occurred due to an abiotic process since removal rates were similar in biological active microcosms and abiotic controls. In filter sand from Brøndby waterworks complete ptaquiloside and caudatoside removal was obtained within the first half hour, probably due to the elevated pH (8.4) caused by the prior softening process.

The filter sand from all investigated waterworks only removed a minor fraction (8-31% after 7 days) of the pyrrolizidine alkaloids (jacobine N-oxide and senecionine N-oxide), and no biological removal was observed.

All the investigated filter sands removed a substantial amount of quinolizidine alkaloids (sparteine and gramine) (between 40% and 100%), and biological processes significantly contributed to the removal. In particular, for filter sand from Regnemark (86% for sparteine), and partly Hvidovre (50% for sparteine), the biological processes were governing the removal. No substantial removal of caffeine was observed (5-17% after 7 days) for any of the investigated filter sands.

A first-order degradation model was applied for fitting the kinetics of phytotoxin removal for ptaquiloside, caudatoside, gramine and sparteine in all investigated waterworks, and degradation rate constants (k_1_) were determined for biologically active microcosms. In most cases the model fitted the data well (R^2^ > 0.90), except for ptaquiloside and caudatoside at Regnemark waterworks (0.85). Removal rate constants for ptaquiloside and caudatoside varied between 1.2 and 32 × 10^-3^ h^-1^ g^-1^, except Brøndby waterworks where due to the high water pH (8.4) degradation happened immediately (removal rate constants > 14000 × 10^-3^ h^-1^ g^-1^). Removal constants of gramine and sparteine varied between 0.1 and 0.6 × 10^-3^ h^-1^ g^-1^. Thus, the observed degradation constants for ptaquiloside and caudatoside were up to two orders of magnitude higher than for gramine and sparteine (Table 6). Filter sand from Regnemark waterworks showed the lowest removal rate constant for ptaquiloside (2.8 × 10^-3^ h^-1^ g^-1^), and was also the waterworks with the lowest water pH (7.3) (Table 3).

**Table 6:** First order degradation constant and R^2^ of removed phytotoxins for five sand filters. “± “ represents standard deviation (n = 2).

	Slangerup			Hvidovre			Brøndby new			Brøndby old			Regnemark		
	**k_1_**	**R^2^**	**n**	**k_1_**	**R^2^**	**n**	**k_1_**	**R^2^**	**n**	**k_1_**	**R^2^**	**n**	**k_1_**	**R^2^**	**n**
PTA	**15±0.006**	**0.95**	**5**	**32±0.004**	**0.96**	**18**	**>14000**	**1**	**18**	**>14000**	**1**	**18**	**2.8±0.02**	**0.85**	**10**
CAU	**1.2±0.001**	**0.96**	**5**	**21±0.002**	**0.98**	**8**	**>14000**	**1**	**18**	**>14000**	**1**	**18**	**10±0.01**	**0.85**	**10**
GRAM	**0.6±0.0001**	**0.94**	**12**	**0.2±0.0001**	**0.93**	**16**	**0.2±0.0001**	**0.95**	**14**	**0.2±0.0001**	**0.93**	**14**	**0.6±0.0001**	**0.93**	**14**
SPAR	**0.1±0.0001**	**0.93**	**12**	**0.1±0.0001**	**0.90**	**18**	**0.1±0.0001**	**0.98**	**14**	**0.2±0.0002**	**0.99**	**14**	**0.6±0.0001**	**0.96**	**14**

Degradation rate constants (k_1_) are presented in 10^-3^ h^-1^ g^-1^ units.

PTA= ptaquilsoide; CAU = caudatoside; GRAM = gramine; SPAR = sparteine.

These variations of the rate constants with changing pH (6-9) are in line with other studies which found that degradation of ptaquiloside followed first order kinetics. According to the Ayala-Luis et al. (2006) model of ptaquiloside degradation, the rate constants of ptaquiloside at 10 °C can be estimated to 2.1 × 10^-2^ h^-1^ to 2.4 × 10^-3^ h^-1^ for the investigated waterworks. The observed rate constants are faster than the predicted rates by about two orders of magnitude. Our results suggest that the existing model (Wu et al., 2021) based on pH and temperature and relevant for hydrolysis in groundwater, does not capture all relevant dynamics in rapid sand filters at waterworks that also contain solids.

### 3.3 Degradation products

We observed fast degradation of ptaquiloside and caudatoside in filter sand from all waterworks included in this study. In particular, at Brøndby waterworks, these compounds were completely hydrolysed within the first half hour, probably due to the high water pH (pH 8.4) (Table 3).

**Fig. 2:**
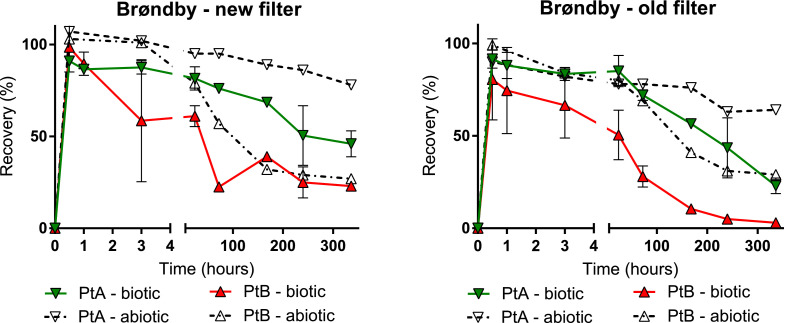
Biotic and abiotic removal of degradation products pterosin B (PtB) and pterosin A (PtA) in sand filters from Brøndby waterworks during 14 days. Mean concentration and standard deviation (error bars) are given as percentage of the initial concentration (C_0_) in biologically active microcosms (duplicates) and abiotic control (single microcosm). ‘Biotic’ refers to microcosms with biologically active filter sand and ‘abiotic’ to microcosms with autoclaved filter sand.

Ptaquiloside and caudatoside hydrolyse to form much more hydrophobic pterosins, pterosin B and pterosin A respectively (Kisielius, 2020; Yamada et al., 2007). Therefore, we investigated the presence of pterosin B and pterosin A in samples from Brøndby waterworks (new and old filter) (Fig. 2). The dissipation of both pterosins were similar in both filters, however with a faster removal of pterosin B compared to pterosin A. Furthermore, the removal in old filter sand was faster than removal in new filter sand. In all cases, the biologically active microcosms removed pterosins faster than autoclaved controls, and removal could therefore partially be ascribed to a biological process. It has previously been established that microorganisms rapidly degrade pterosin B (Skourti-Stathaki et al., 2016). Observed degradation of pterosins was much slower in comparison to ptaquiloside and caudatoside.

Additionally, the degradation intermediate dienone was detected during degradation of ptaquiloside, and was present in the microcosms until the end of the study (but not quantified). A dienone is an unstable degradation compound, and is assumed to be the ultimate agent responsible for carcinogenicity with the cyclopropyl group reacting with amino acids and DNA, and causing mutations (da Costa et al., 2012). Degradation of dienone subsequently leads to formation of non-carcinogenic pterosin B (Figure S7). Thus, even though carcinogenic ptaquiloside was rapidly degraded in filter sands, toxic degradation products were still present.

We did not screen for degradation products of alkaloids in all experiments, but the transformation of jacobine N-oxide and senecionine N-oxide to corresponding free base form was observed and quantified at Regnemark waterworks (Fig. 1, E and F). Observed free base forms of jacobine and senecionine are the primary degradation products of pyrrolizidine alkaloids N-oxide (Chou et al. 2003; Hama, 2020), whereas the transformation of jacobine N-oxide and senecionine N-oxide to retronecine (second degradation product, Figure S6) was not detected, which could be due to short half-life of retronecine (Hama, 2020). For gramine and sparteine it was not possible to detect formation of any degradation products, although the samples were screened for possible products (Table S2).

## 4 Variations of filter sand from all waterworks

### 4.1 Physicochemical properties

To characterize filter sand material, we determined particle size distribution, specific surface areas and content of Fe, Mn, C and N, and the results showed that specific surface areas correlated highly with the content of metal oxides as well as C and N content in all filters (Table 4). Filter sand from Regnemark had the largest surface area and the highest carbon and metal oxide contents (71 mg Fe g^-1^ and 65 mg Mn g^-1^). This filter sand also showed the fastest degradation of phytotoxins (Table 5) among the investigated filters. At Brøndby waterworks, better removal of pterosins was observed for the old filter (Fig. 2) with higher metal oxide content and larger surface area in comparison to the new filter. In addition, only in the new filter sand from Brøndby, transformation of jacobine N-oxide and senecionine N-oxide to free base forms was observed in lower extent in abiotic samples (60% present after 14 days, Figure S3), where very little metal oxides coating were present.

Presence of metal oxides could increase sorption processes and manganese oxides are one of the most active metal oxide catalysts, which are shown to be excellent agents in the oxidation of various compounds (e.g. toxic volatile organic compounds) (Liao et al., 2017). Furthermore, removal of other organic contaminants in filter sand (e.g. the herbicide glyphosate) was mainly attributed to adsorption in relation to the presence of metal oxides (Hedegaard et al., 2014). Metal oxides coatings on filter sand surfaces can also positively affect the colonization and activity of microbiological communities (Gülay et al., 2014) and hence enhance biological removal.

### 4.2 DNA analyses of filter sand

**Fig. 3:**
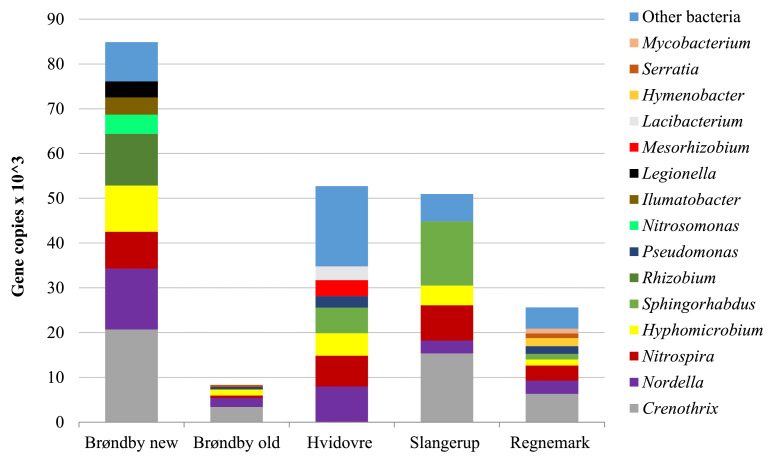
Dominating bacteria genera (>500 gene copies) in sand filter material from five investigated waterworks before microcosms experiments were performed. The bars show absolute numbers of bacteria gene copies in each of the five samples.

DNA was extracted from all investigated sand filters in order to characterize bacteria communities. The new filter at Brøndby waterworks contained the highest number of gene copies (84,885), while the lowest number was detected at the old filter (Fig. 3). We characterized 18 dominating bacteria genera, which each constituted more than four percent of the total bacterial DNA. Dominating genera in the filter sand material was *Crenothrix* and *Nordella*, followed by *Nitrospira, Hyphomicrobium* and *Sphingorhabdus*. The group labelled *other bacteria* in this study represents all bacteria that constituted below four percent of the total bacterial DNA, but still above the cut-off value of 500 number of sequences. At Hvidovre waterworks, other bacteria make a significant proportion of 34%. Overall, bacteria present in the investigated sand filters differed between waterworks, but could not apparently explain the observed differences in removal efficiencies of the sand filters. The majority of eukaryotic sequences in each investigated sand filter belongs to phyla of *Amoebozoa, Cercozoa* and *Ciliophora* (for total numbers of bacterial and eukaryotic gene copies see Figure S5).

The bacterial community in filters may be connected to its main functions (oxidation of iron, manganese, ammonium and methane) (Albers et al., 2015). *Crenothrix* belongs to the family *Methylococcaceae* which are Type I methanotrophs that obtain their carbon and energy from methane oxidation (Albers et al., 2015). *Nitrospira* is a well known nitrite-oxidizing bacteria in groundwater (Fowler et al., 2018), while a recent study correlated the genus *Nordella* with Mn(II) oxidation in rapid sand filters (Zhao et al., 2020). The genus *Hyphomicrobium* was proposed as a key Mn(II) oxidizer (Albers et al., 2015). Organic trace contaminants can be degraded either by primary metabolism, where microorganisms utilize them as sources of carbon and possibly also nitrogen, phosphorus, or sulphur (Alexander, 1999), or by co-metabolism where contaminants are transformed by enzymes without being used as an energy source (Vickers, 2017). Other studies have shown that co-metabolism by methane oxidizing bacteria in rapid sand filters are relevant for degradation of the pesticide bentazone (Hedegaard et al., 2020). Biological removal was only observed for gramine and sparteine and was primarily of importance at Regnemark waterworks. At Slangerup waterworks, which receives the water with highest concentrations of methane (Table 3) no biological removal occurred (Table 5) and thus there were no indications that methanotrophs governed the removal of phytotoxins. Further studies are needed to determine which biological process removed the phytotoxins.

**Fig. 4:**
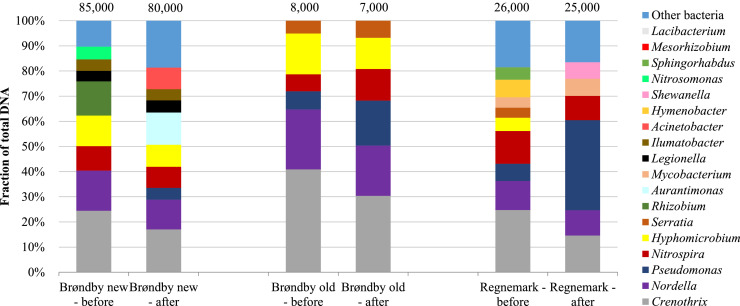
Dominating bacteria genera (>500 gene copies) in filter sand from Regnemark waterworks and Brøndby waterworks (new and old filter) before and after the experiment. Total numbers of gene copies per sample are presented above the columns. The bars show the relative abundance of different bacteria in the samples.

DNA from microcosms with filter sands from Brøndby and Regnemark waterworks was analysed before and after the experiments. During the experiments the bacteria community changed similarly in all three sand filters (Fig. 4). Hence, the most abundant genera decreased (e.g. *Crenothrix* and *Nordella*), while the fraction of heterotrophic bacteria genera increased (e.g. *Pseudomonas*). Hence, for the filter sand from Regnemark waterworks, which showed the highest biological removal (Table 5), the fraction of heterotrophs increased from 7% to 36% during the experiment. However the increase in heterotrophic bacteria may merely be due to regrowth in the stagnant water (Yi et al., 2019), since the increase was observed in all three investigated microcosms. To be able to make stronger link between the microbial community members and phytotoxin degradation, it would be essential to perform more rigorous replication as well as other analyses targeting activity and/or functionality such as stable-isotope probing (SIP) and mRNA.

### 4.3 Perspective

To our knowledge, this is the first investigation on phytotoxin degradation in filter sand from rapid sand filters. We found that ptaquiloside and caudatoside were removed in filter sand with first-order rate constants of >1.2 × 10^-3^ h^-1^ g^-1^, while rate constants for removal of gramine and sparteine varied between 0.1 and 0.6 × 10^-3^ h^-1^ g^-1^, showing a substantial removal potential of phytotoxins in filter sand. However, to be able to quantify degradation, we applied phytotoxins at initial concentrations of 300 µg L^-1^, which is higher than concentrations found in the environment (Günthardt et al., 2020; Hama, 2020; Kisielius et al., 2020a; Nanusha et al., 2020; Skrbic et al., 2021), but is expected to cover extreme environmental conditions. Removal kinetics of the investigated compounds could be different at lower concentrations in real rapid sand filters, which are flow-through systems. However, other studies have found similar removal rates of pesticides in batch experiments with filter sand from waterworks, as we found for phytotoxins (e.g. herbicide mecoprop (MCPP): 1.6 × 10^-3^ h^-1^ g^-1^), even though the initial pesticide concentration was 0.2 µg L^-1^ (Hedegaard et al., 2014; Papadopoulou et al., 2018). The norsesquiterpene glycosides are unique structures and their instability mainly derives from their ready hydrolysis by which the compounds transform to aromatic products which are much more stable than the parent compounds. The alkaloids share the aromatic backbone with the pesticides mentioned, but with the N substitutions stabilizing the structures instead of halogens for the pesticides.

Since the contact time of the investigated sand filters was up to 72 min, and a substantial removal of the investigated phytotoxins occurred within the first hour of the experiments (Fig. 1; Fig. 2), there is a potential for phytotoxins to be removed in a full-scale system. However, this needs to be further investigated in flow-through systems, at environmentally relevant concentrations.

Pellet softening of drinking water was recently implemented for the first time in Denmark (HOFOR 2017) and results in an increased pH (8.4) of the water. Degradation kinetics of bracken toxins increases at pH > 7 (Ayala‐Luis et al., 2006), and since pellet softening (and hence elevated pH) is already implemented before the sand filters at Brøndby waterworks, this explains why degradation of bracken toxins occurred immediately in filter sand from this waterworks. The presence of the carcinogenic dienone intermediate was detected at the high water pH, suggesting that drinking water quality might remain compromised. In addition, pellet softening limits formation of new coatings on the filter sand, since it removes metals upstream of the filters. This could decrease the removal potential for various micropollutants, since it reduces coatings of manganese oxides which stimulate degradation of various organic compounds (Liao et al., 2017). Mineral coating might affect jacobine N-oxide and senecionine N-oxide transformation to free base forms, since transformation in autoclaved controls with new filter sand from Brøndby waterworks, was substantially lower compared to other filter sands.

The microcosms set-up used in this study proved to be a robust and fast approach to test removal of phytotoxins in drinking water systems and could be applied to a broader group of phytotoxins. This study also found that the suggested predictive models by some studies on phytotoxins stability (Ayala‐Luis et al., 2006) are not directly applicable to actual waterworks, as predicted removal rates were orders of magnitudes lower than observed in this study.

## 5 Conclusions

We have demonstrated a removal potential of phytotoxins in filter sand obtained from biological rapid sand filters for the first time. To screen the removal potential of several different phytotoxins in different filter sands the investigations were carried out as batch experiments. Phytotoxins were applied at initial concentrations of 300 µg L^-1^ to test worst-case scenario. We found that filter sand from five different rapid sand filters showed similar removal potential of the investigated phytotoxins.

All filter sands removed ptaquiloside and caudatoside completely from the water phase by an abiotic process. This removal depended on pH and occurred immediately at a waterworks where pellet softening was implemented prior to rapid sand filtration and pH thus reached 8.4. During degradation of ptaquiloside and caudatoside we observed formation and subsequent removal of their hydrolysis products pterosin B and pterosin A, while toxic intermediates dienones were also formed and maintain the water toxicity. In contrast, biological degradation governed the removal of the quinolizidine alkaloids, sparteine and gramine. Differences between bacterial communities could not immediately explain different removal in the filter sands. However, along with biological degradation, we observed an increase in the presence of heterotrophic bacteria in filter sands. Waterworks with the highest removal potential were characterized by high contents of iron and manganese oxide coatings and large sand filter specific surface areas.

The investigated filter sand removed certain phytotoxins, namely bracken toxins and quinolizidine alkaloids. Hence, if these phytotoxins enter wells used for drinking water production, they might be removed in the already existing groundwater treatment. However, other groups of phytotoxins (e.g. pyrrolizidine alkaloids) are stable in filter sand, which calls for further investigations involving more advanced treatment processes that could remove these compounds.

## Authors' contributions

Conceptualization: N.S.M., J.H., B.W.S., H.C.B.H., L.H.R., A.-K.P., S.C.B.C. and M.J.H. Investigations: N.S.M. Data curation and visualization: N.S.M. and M.J.H. Funding acquisition: A.-K.P., B.W.S., H.C.B.H. and L.H.R. Supervision: N.S.M., H.C.B.H., L.H.R., A.-K.P., S.C.B.C., and M.J.H. Writing-original draft: N.S.M. Writing-Review and editing: N.S.M., J.H., H.C.B.H., L.H.R., S.C.B.C. and M.J.H. All authors read and approved the final manuscript.

## Funding

This project was funded by the European Union's 10.13039/100010661Horizon 2020 research and Innovation Programme under the Marie Sklodowska-Curie grant agreement No. 722493 (NaToxAq).

## Declaration of Competing Interest

No conflict of interest to declare.

## Appendix Supplementary materials

Image, application 1
