# Supplementary material for: Removal of phytotoxins in filter sand used for drinking water treatment
Source: Water Res. 2021 Oct 15;205:117610. doi: 10.1016/j.watres.2021.117610 (PMC8556162; doi:10.1016/j.watres.2021.117610)
Supplement: Supplementary file 1 [file mmc1.docx]

Supplementary material

**Removal of phytotoxins in filter sand used for drinking water treatment**

Natasa Skrbic Mrkajic, Jawameer Hama, Bjarne W. Strobel, Hans Christian Bruun Hansen, Lars Holm Rasmussen_,_ Ann-Katrin Pedersen, Sarah C. B. Christensen, Mathilde J. Hedegaard

**LC-MS analyses of bracken toxins**

Quantification of ptaquiloside and caudatoside were performed by Agilent 1260 Infinity HPLC System equipped with Agilent 6130 Single Quadrupole mass spectrometer by the method described by Kisielius *et al*. (2020b). The chromatographic separation was performed using Agilent Poroshell 120 EC-C18 column. Mobile phase A (water) and B (acetonitrile) were used, both with 0.1% v/v formic acid in gradient elution mode. Injection volume of samples was 10 μL and flow rate was 1 mL L^-1^. The instrumental limits of detection (LOD) and quantification (LOQ) were: 0.22/0.68 µg L^-1^ (ptaquiloside), 0.26/0.78 µg L^-1^ (caudatoside), 0.03/0.09 µg L^-1^ pterosin B and 0.02/0.05 µg L^-1^ pterosin A. The dienone of ptaquiloside was tentatively identified from the ESI MS spectra of the eluting peak at 4.40 minutes (dienone + H+: m/z 219.1; dienone - H2O: m/z 201.1). No analytical standards are available to validate the compound identity (Kisielius et al., 2021).

In the LC-MS following set of parameters was used: Cone voltage (CV): 3,000 V; fragmentor voltage 110 V; drying gas flow: 13 L min^-1^; drying gas temperature: 350 °C; nebulizer pressure: 40 psi. The mass fragments were monitored in single and total ion mode. The retention times and mass-to-charge ratios are listed in Table S1. LOD was calculated as 3.3 times the SDintercept/slope of the calibration curves. LOQ was calculated as 10 times the SDintercept/slope of the calibration curves. OpenLab CDS ChemStation was used for data acquisition and processing (Agilent, USA).

Table S1. LC-MS parameters for selected norsesquiterpene glycosides and their degradation products: retention time (t_R_), mass-to-charge ratio (m/z) and CV.

|  | **t_R_ (min)** | **m/z** |
| --- | --- | --- |
| Ptaquiloside | 3.67 | 219.1 |
| Caudatoside | 3.40 | 249.1 |
| Pterosin B | 4.61 | 219.1 |
| Pterosin A | 4.01 | 249.1 |

**LC-MS/MS analyses of alkaloids**

For quantification of alkaloids, samples were analysed on a Waters Acquity UPLC I-Class module, equipped with Acquity UPLC HSS C18 column (2.1 × 100 mm, 1.8 μm) (Waters, Milford) by the method described by Hama and Strobel (2019, 2020b). Waters Xevo TQD triple quadrupole mass spectrometer (MS/MS) was operated with electrospray ionization in positive ion mode. Mobile phases of A (water + 0.1% FA) and B (MeOH + 0.1% FA) were used for gradient elution. Sample volume was 2.5 μL, and column flow rate 0.20 mL L^-1^. LOD and LOQ were: 4.7/6.2 µg L^-1^ (jacobine N-oxide), 2.60/4.5 µg L^-1^ (jacobine), 5.6/9.5 µg L^-1^ (senecionine N-oxide), 5.42/9.70 µg L^-1^ (senecionine), 0.21/0.73 µg L^-1^ (sparteine) 0.2/0.38 µg L^-1^ (gramine) and 0.005/0.009 µg L^-1^ (caffeine). Detection was performed using full scans and multiple reaction monitoring (MRM) for all alkaloids. More details about MS settings is provided in supplementary material (SM) Table S1 and Table S2.

In the MS/MS, the ion traces were obtained for apex retention time (tR) ± 0.15 min, the corresponding cone voltages and collision energies (CE) are listed in Table S2. The desolvation temperature was 600°C, the desolvation gas flow 1000 L h^-1^, the source temperature 150 °C, the cone gas flow 20 L h^-1^, and the capillary voltage 3.5 kV. The LOD and LOQ were calculated in water, using 7 injections of 25 µg L^-1^ standards solution, then they were calculated as 3 and 10 times respectively the standard deviation of peak areas divided by the slope of the calibration curve for each standard. MassLynx™ version 4.1 was used for data acquisition and processing (Waters, Milford, USA).

Table S2. LC-MS/MS parameters for selected alkaloids: retention time (t_R_), precursor ion, product ion, CV and CE.

|  | **t_R_ (min)** | **Precursor**  **ion (m/z)** | **Product**  **ion (m/z)** | **Cone voltage (V)** | **Collision energy (eV)** |
| --- | --- | --- | --- | --- | --- |
| Gramine | 5.62 | 175.1 | 130.06 | 15 | 15 |
| Sparteine | 6.98 | 235.2 | 98.09 | 30 | 35 |
| Jacobine | 7.18 | 352.2 | 120.08, 138.09 | 40 | 40 |
| Jacobine N-oxide | 7.89 | 368.2 | 120.08, 138.09 | 40 | 40 |
| Senecionine | 11.03 | 336.2 | 120.08, 138.09 | 20 | 30 |
| Senecionine N-oxide | 11.51 | 352.2 | 120.08, 138.09 | 40 | 30 |
| Caffeine | 9.51 | 195.1 | 109.02 | 25 | 35 |

To avoid matrix effect as observed using MeOH and water for preparing the standard solutions, quantification took place using matrix-matched calibrations, produced freshly for each batch of samples. Matrix-matched calibration curves were obtained by preparing the standard solutions in the water samples that were used in each of the sand filter experiments.

**Characterisation of filter sand material**

Filter sand samples were kept at - 20 °C until physicochemical analyses. Prior to analysis, oven drying of the wet samples was performed in 50 mL polyethylene tubes at 60 °C. Brunauer–Emmett–Teller (BET) specific surface area (SSA) of the filter sand materials were determined by Micromeritics TriStar II 3020 following degassing at 40 °C in a vacuum over night using N_2_. Particle size distribution of filter sand was performed by sieving. Fe and Mn was extracted from the filter sand materials by extraction of 0.25 g of filter sand material with 25 mL of 4 M HCl and boiled for 30 min, followed by metal determination by atomic absorption spectroscopy. The total contents of C and N in the filter sand materials was determined using a C/N analyser (Elementar Vario Macro Cube). The sample was prepared by mixing 10-15 mg of sample with 10-15 mg tungsten. Sulfanilamide was used as standard.

**DNA sequencing data**

DNA sequencing data generated in this study are publicly available in European Nucleotide Archive (ENA). Accession numbers to ENA and more details about samples are provided in Table S3.

Table S3. DNA sequencing data generated in this study with the accession numbers to the European Nucleotide Archive (ENA).

| **#** | **Accession number** | **Alternative accession number** | **Sample description** | **Label in Figure 3** | **Label in Figure 4** |
| --- | --- | --- | --- | --- | --- |
| 1 | ERS7262687 | SAMEA9539915 | HOFOR-2-slangerup-sand-sandfilter | Slangerup | - |
| 2 | ERS7262688 | SAMEA9539916 | HOFOR-2-hvidovre-sand-sandfilter | Hvidovre | - |
| 3 | ERS7262689 | SAMEA9539917 | HOFOR-2-broendby2-before-sandfilter | Brøndby new | Brøndby new before |
| 4 | ERS7262690 | SAMEA9539918 | HOFOR-2-broendby3-before-sandfilter | Brøndby old | Brøndby old before |
| 5 | ERS7262691 | SAMEA9539919 | HOFOR-2-broendby2-after-sandfilter | - | Brøndby new after |
| 6 | ERS7262692 | SAMEA9539920 | HOFOR-2-broendby3-after-sandfilter | - | Brøndby old after |
| 7 | ERS7262693 | SAMEA9539921 | HOFOR-2-regnemark-before-sandfilter | Regnemark | Regnemark before |
| 8 | ERS7262694 | SAMEA9539922 | HOFOR-2-regnemark-after-sandfilter | - | Regnemark after |


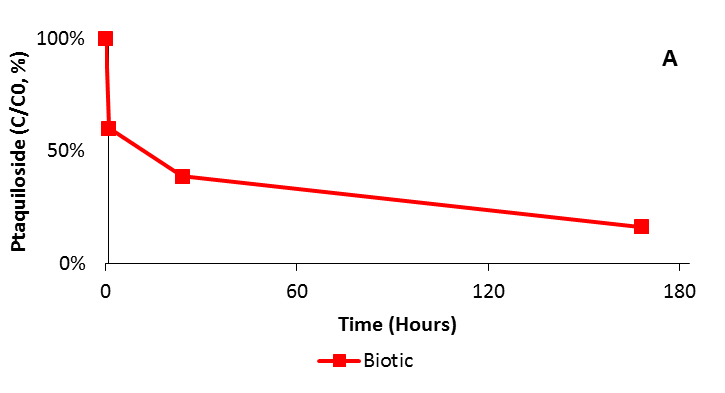

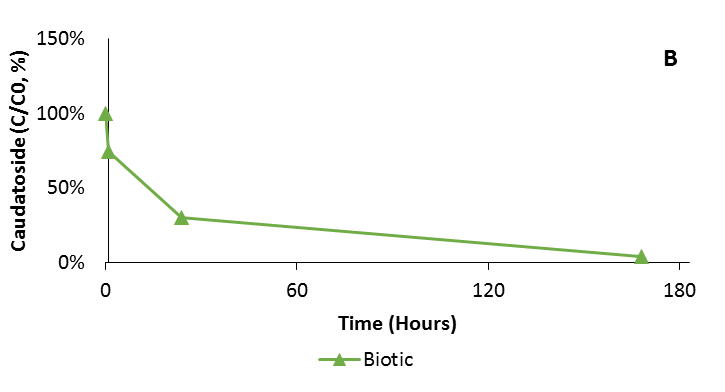


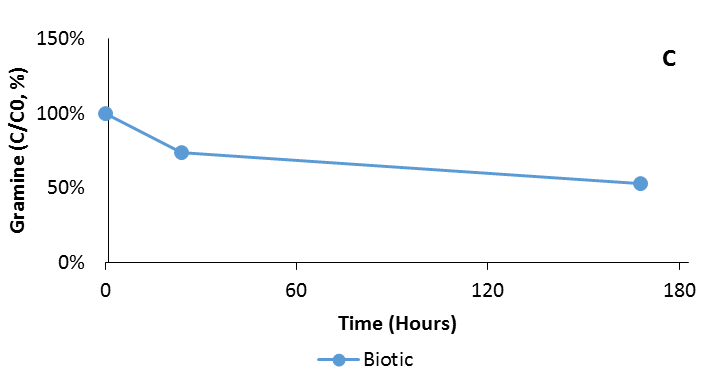

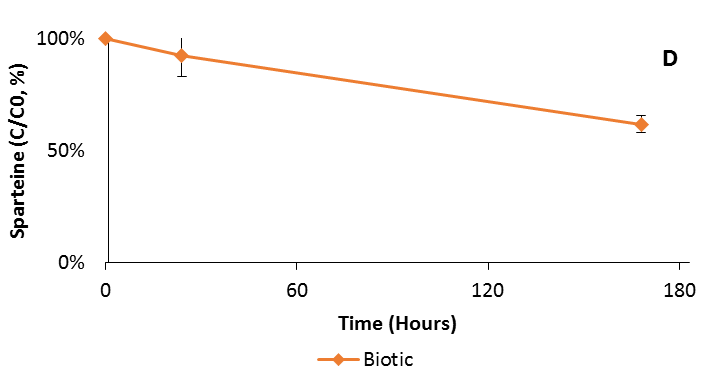


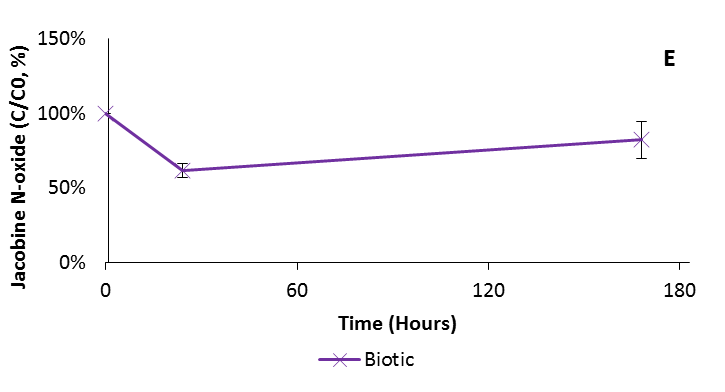

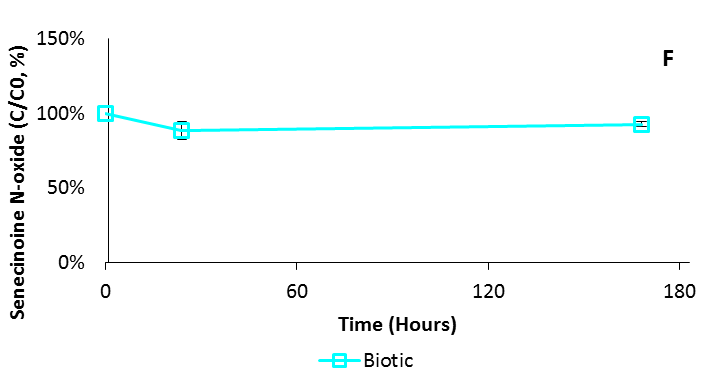


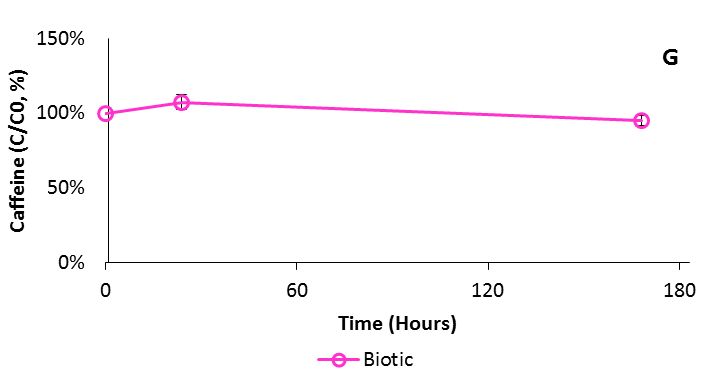


Figure S1. Dissipation of seven phytotoxins in microcosms using sand filter material from Slangerup waterworks over a period of 7 days (at 10 °C). Mean concentration and standard deviation (error bars) are given as percentage of the initial concentration (C­_0_, 300 µg L^-1^) in biologically active microcosms (duplicates). ‘Biotic’ refers to microcosms with biologically active filter sand.


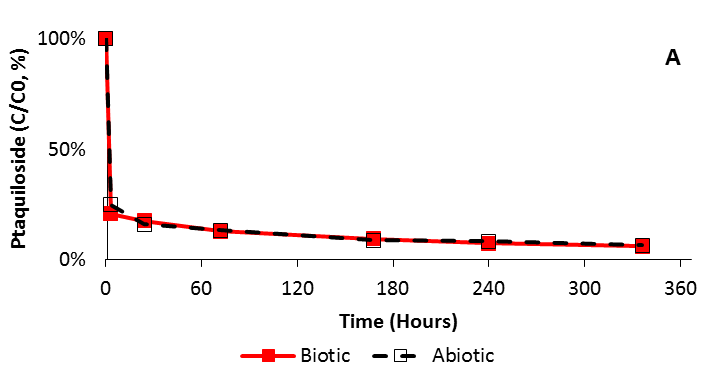

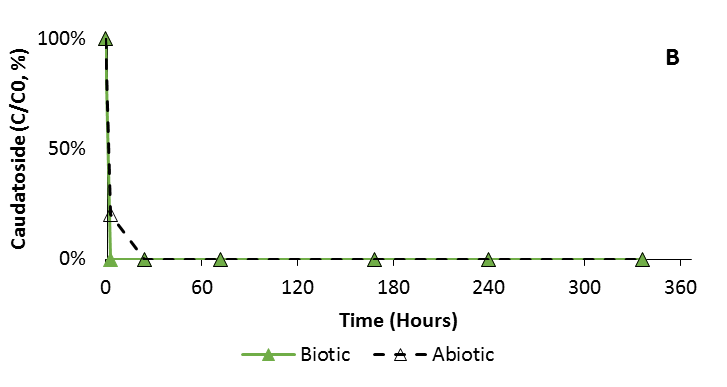

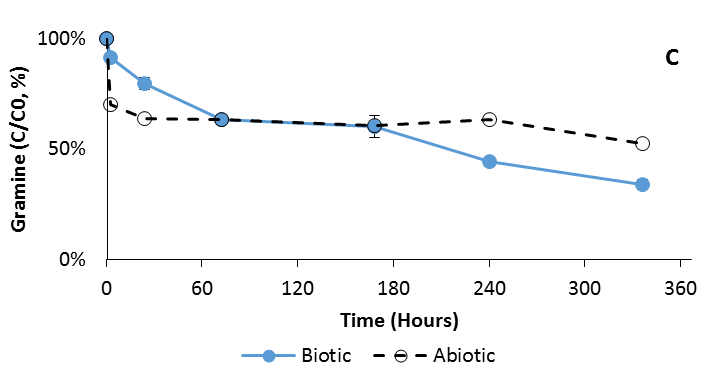

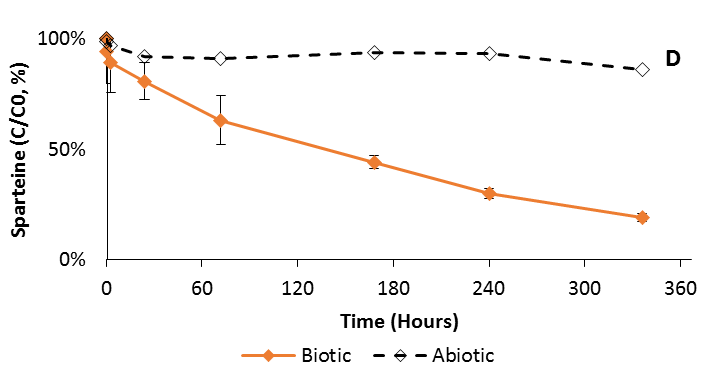

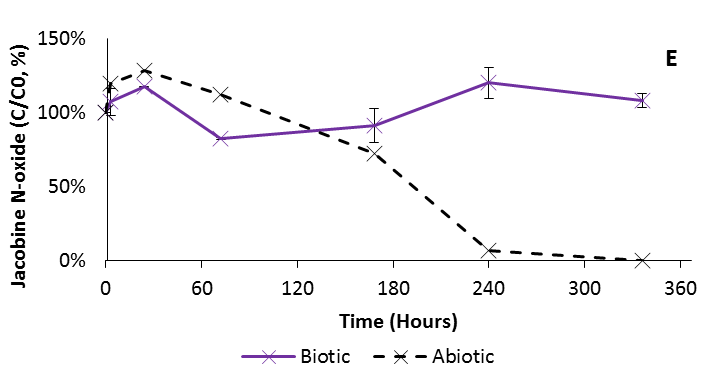

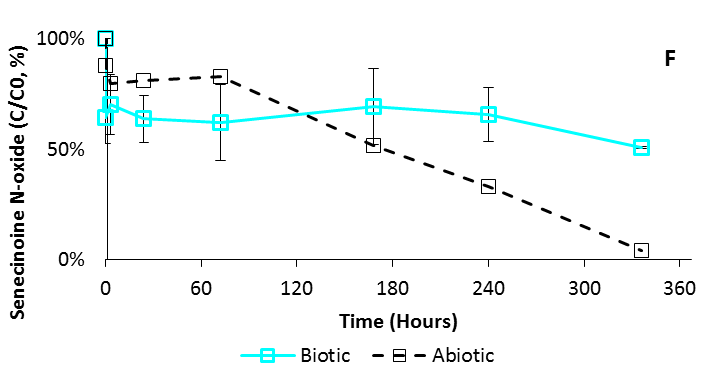

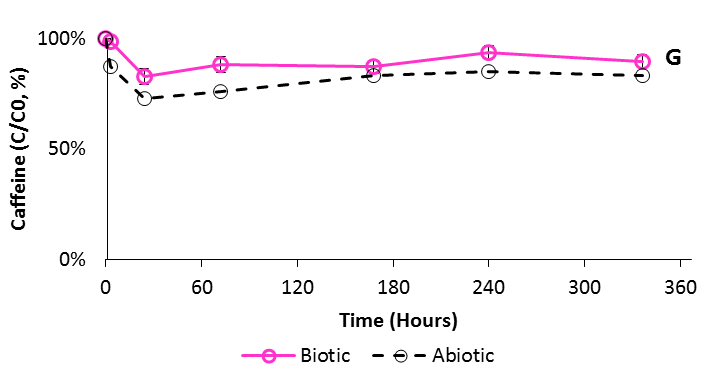


Figure S2. Dissipation of seven phytotoxins in microcosms using sand filter material from Hvidovre waterworks over a period of 14 days (at 10 °C). Mean concentration and standard deviation (error bars) are given as percentage of the initial concentration (C­_0_, 300 µg L^-1^) in biologically active microcosms (duplicates) and abiotic control (single microcosm). ‘Biotic’ refers to microcosms with biologically active filter sand and ‘abiotic’ to microcosms with autoclaved filter sand.

.


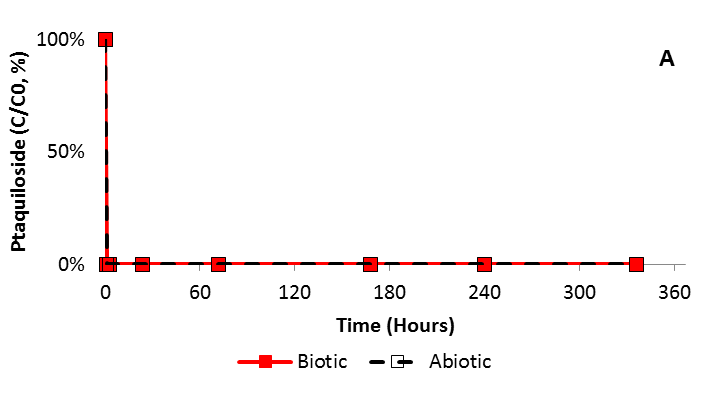

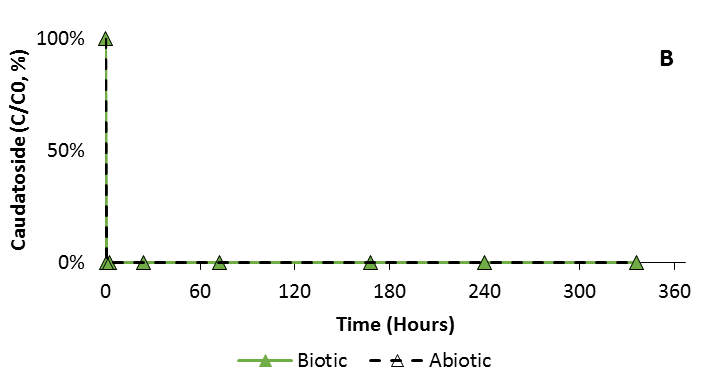

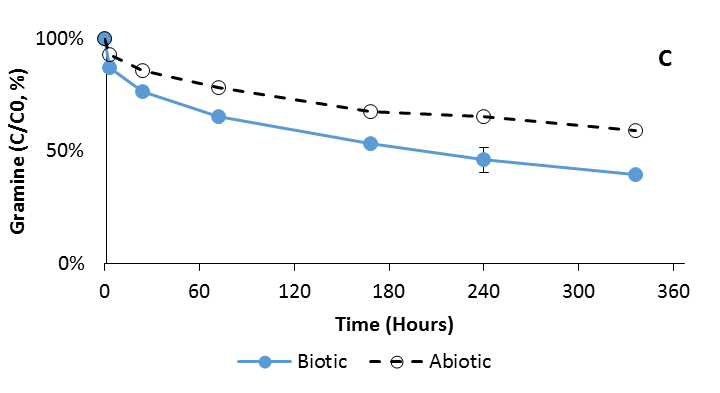

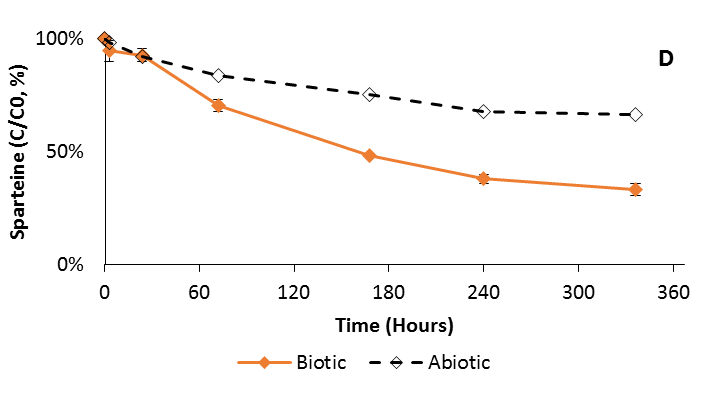

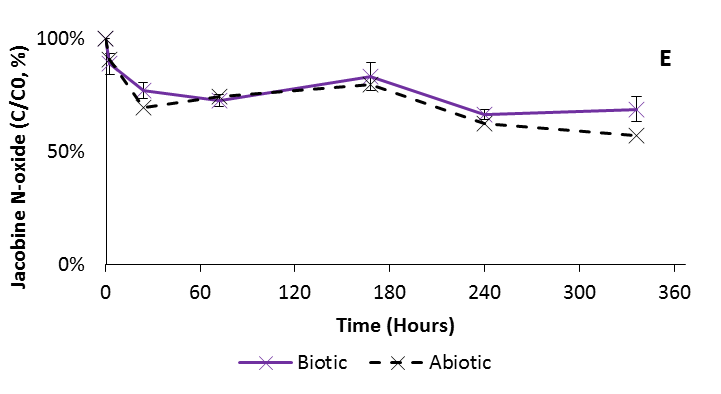

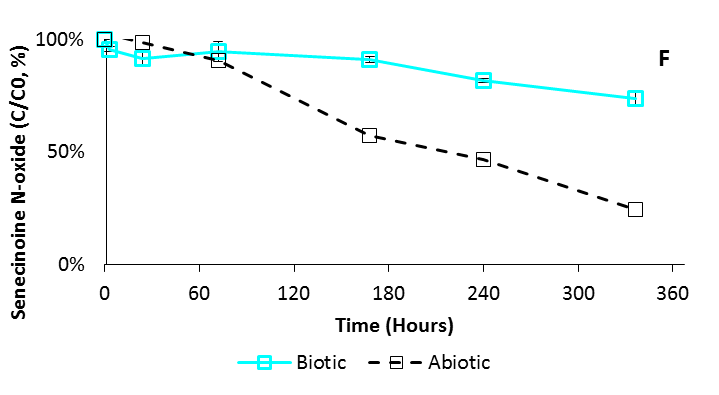

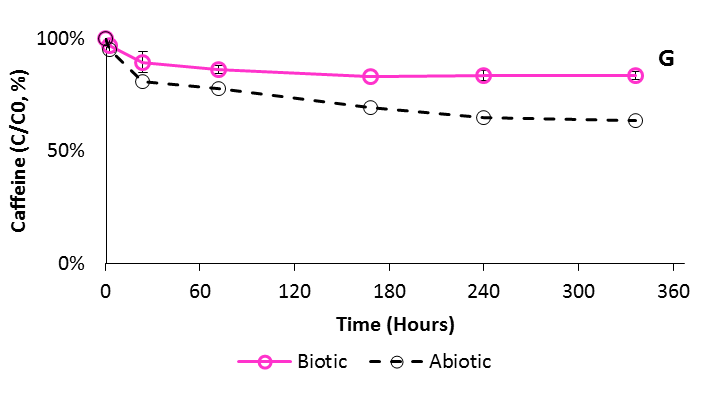


Figure S3. Dissipation of seven phytotoxins in microcosms using sand filter material from Brøndby waterworks (new filter) over a period of 14 days (at 10 °C). Mean concentration and standard deviation are given as percentage of the initial concentration (C­_0_, 300 µg L^-1^) in biologically active microcosms (duplicates) and abiotic control (single microcosm). ‘Biotic’ refers to microcosms with biologically active filter sand and ‘abiotic’ to microcosms with autoclaved filter sand.


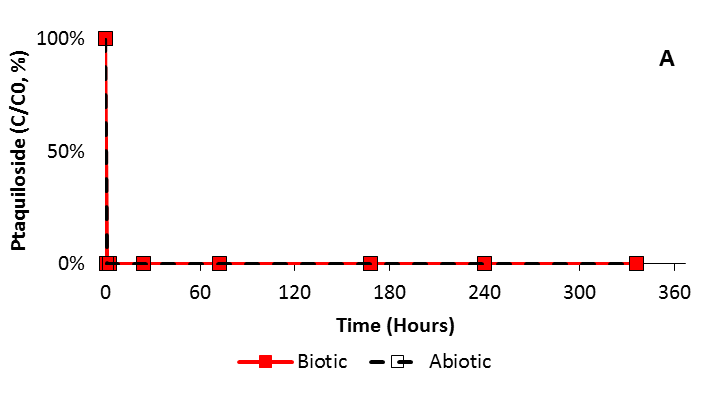

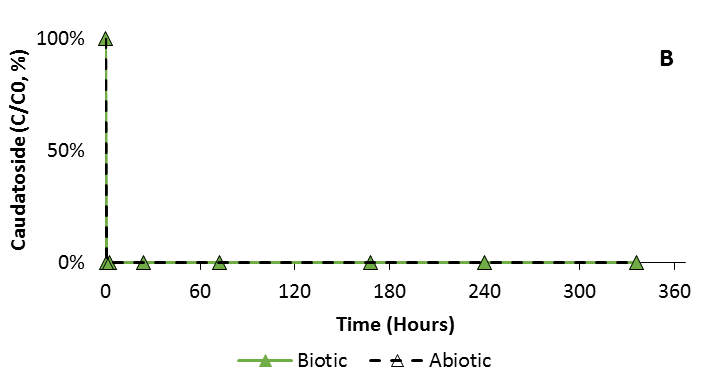

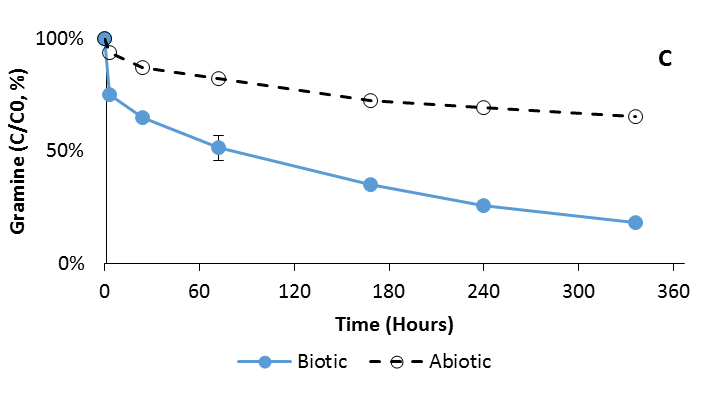

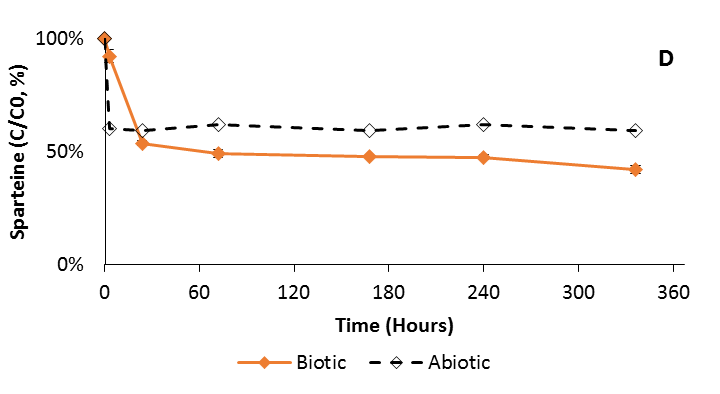

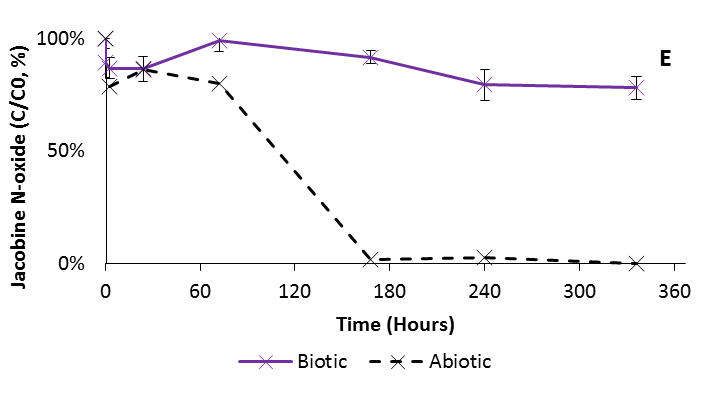

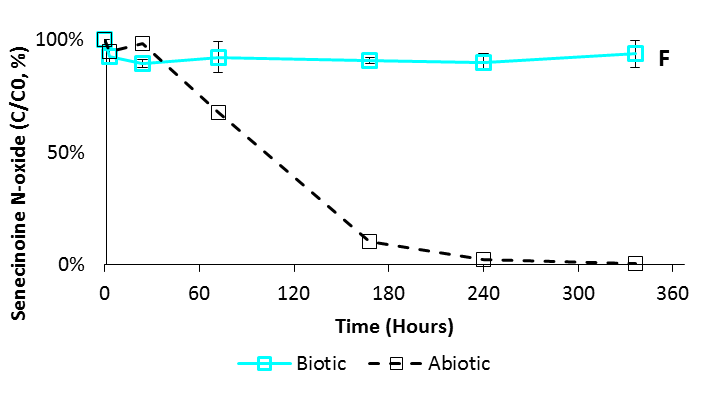

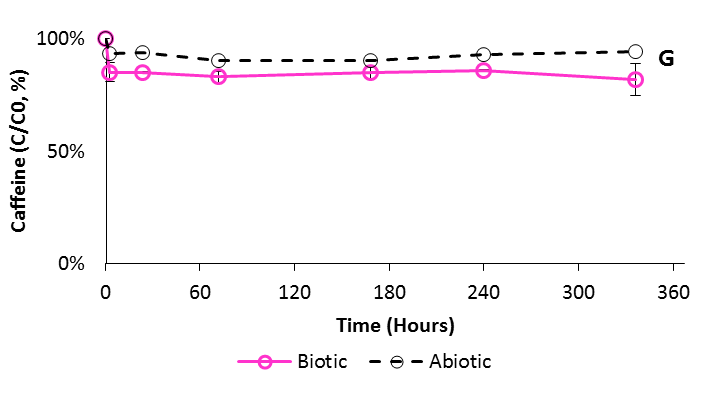


Figure S4. Dissipation of seven phytotoxins in microcosms using sand filter material from Brøndby waterworks (old filter) over a period of 14 days (at 10 °C). Mean concentration and standard deviation are given as percentage of the initial concentration (C­_0_, 300 µg L^-1^) in biologically active microcosms (duplicates) and abiotic control (single microcosm). ‘Biotic’ refers to microcosms with biologically active filter sand and ‘abiotic’ to microcosms with autoclaved filter sand.


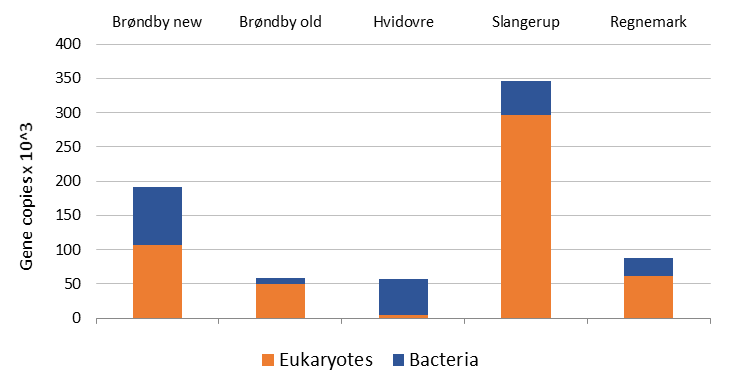


Figure S5. Absolute number of bacteria and eukaryote sequences at five investigated-waterworks.


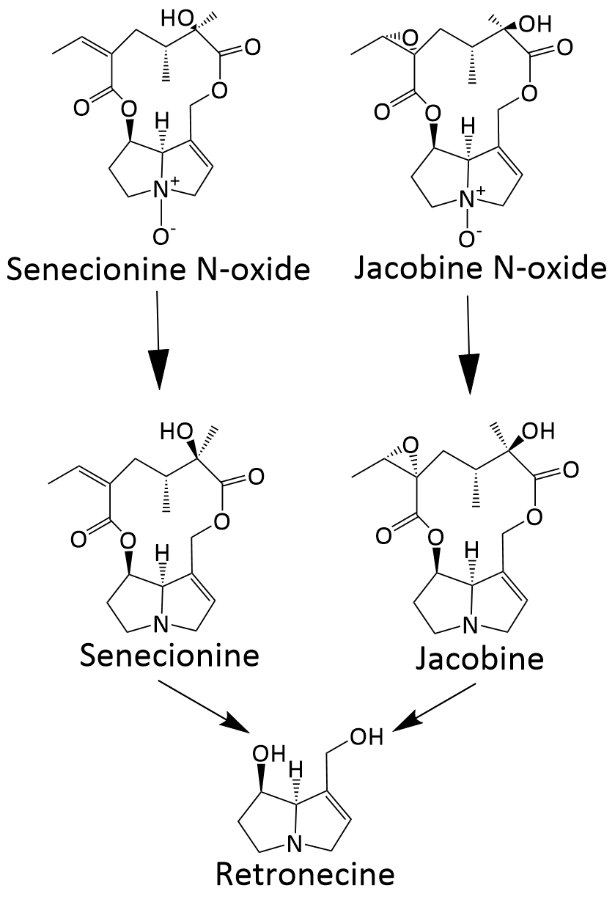


Figure S6. Possible degradation pathways of main metabolites; senecionine N-oxide and jacobine N-oxide, to form the corresponding free base pyrrolizidine alkaloids, senecionine and jacobine (primary degradation product), and then retronecine (secondary degradation product) during removal experiments.


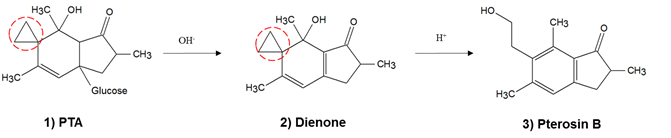


Figure S7. Hydrolysis of PTA (1) to dienone (2) and PtB (3).
